# Downregulation of SLC27A6 by DNA Hypermethylation Promotes Proliferation but Suppresses Metastasis of Nasopharyngeal Carcinoma Through Modulating Lipid Metabolism

**DOI:** 10.3389/fonc.2021.780410

**Published:** 2022-01-03

**Authors:** Xuemin Zhong, Yanping Yang, Bo Li, Pan Liang, Yiying Huang, Qian Zheng, Yifang Wang, Xue Xiao, Yingxi Mo, Zhe Zhang, Xiaoying Zhou, Guangwu Huang, Weilin Zhao

**Affiliations:** ^1^ Department of Otolaryngology-Head and Neck Surgery, First Affiliated Hospital of Guangxi Medical University, Nanning, China; ^2^ Key Laboratory of Early Prevention and Treatment for Regional High Frequency Tumor (Guangxi Medical University), Ministry of Education, Nanning, China; ^3^ Guangxi Key Laboratory of High-Incidence-Tumor Prevention & Treatment (Guangxi Medical University), Nanning, China; ^4^ Department of Radiotherapy, First Affiliated Hospital of Guangxi Medical University, Nanning, China; ^5^ Life Science Institute, Guangxi Medical University, Nanning, China; ^6^ Department of Research, Affiliated Tumor Hospital of Guangxi Medical University, Nanning, China

**Keywords:** nasopharyngeal carcinoma, SLC27A6, proliferation, metastasis, fatty acid metabolism

## Abstract

Lipid is the building block and an important source of energy, contributing to the malignant behavior of tumor cells. Recent studies suggested that lipid droplets (LDs) accumulations were associated with nasopharyngeal carcinoma (NPC) progression. Solute carrier family 27 member 6 (SLC27A6) mediates the cellular uptake of long-chain fatty acid (LCFA), a necessary lipid component. However, the functions of SLC27A6 in NPC remain unknown. Here, we found a significant reduction of SLC27A6 mRNA in NPC tissues compared with normal nasopharyngeal epithelia (NNE). The promoter methylation ratio of SLC27A6 was greater in NPC than in non-cancerous tissues. The demethylation reagent 5-aza-2’-deoxycytidine (5-aza-dC) remarkably restored the mRNA expression of SLC27A6, suggesting that this gene was downregulated in NPC owing to DNA promoter hypermethylation. Furthermore, SLC27A6 overexpression level in NPC cell lines led to significant suppression of cell proliferation, clonogenicity *in vitro*, and tumorigenesis *in vivo*. Higher SLC27A6 expression, on the other hand, promoted NPC cell migration and invasion. In particular, re-expression of SLC27A6 faciliated epithelial-mesenchymal transition (EMT) signals in xenograft tumors. Furthermore, we observed that SLC27A6 enhanced the intracellular amount of triglyceride (TG) and total cholesterol (T-CHO) in NPC cells, contributing to lipid biosynthesis and increasing metastatic potential. Notably, the mRNA level of SLC27A6 was positively correlated with cancer stem cell (CSC) markers, CD24 and CD44. In summary, DNA promoter hypermethylation downregulated the expression of SLC27A6. Furthermore, re-expression of SLC27A6 inhibited the growth capacity of NPC cells but strengthened the CSC markers. Our findings revealed the dual role of SLC27A6 in NPC and shed novel light on the link between lipid metabolism and CSC maintenance.

## Introduction

Nasopharyngeal carcinoma (NPC) is a malignancy arising from the mucosa of the nasopharynx, which is predominantly associated with Epstein-Barr virus (EBV) latent infection (1, 2). The geographical distribution of NPC is unbalanced worldwide, relatively rare in Western countries, while particularly prevalent in southern China and Southeast Asia. In endemic areas, the pathological type of NPC is mostly non-keratinizing and poorly-differentiated squamous cell carcinoma (2, 3). Genetic factors, exposure to carcinogens, and EBV latent infection are the main etiologies of NPC (1, 4). Besides, epigenetic modification, especially DNA promoter hypermethylation, has played a critical role in the NPC tumorigenic process. To date, various important tumor suppressor genes (TSGs) have been identified in NPC, downregulated by DNA promoter hypermethylation, such as RASSF2A, CDH4, RERG (5–7).

As a hallmark of cancer, metabolic reprogramming has been demonstrated to facilitate tumorigenesis (8, 9). Fatty acids (FAs) are composed of lipids and provide sufficient energy resources for cancer cells. Mounting evidence showed that *de novo* FA synthesis was highly upregulated in multiple cancers (9, 10). In hepatocellular carcinoma, fatty acid synthase (FASN) was overexpressed in high metastatic hepatocellular carcinoma cells, and inhibition of FASN contributed to hepatocellular carcinoma metastasis suppression (11). Indeed, lipid metabolism disorders are essential for cancer cell proliferation, motility, differentiation, and metastasis (12, 13). Recent evidence indicated that lipid droplets (LDs) excessed in NPC cells, and EBV latent infection product LMP2A enhanced this phenomenon in NPC (14). In addition, LMP2A mediated a series of metabolism-associated genes shift and rewired lipid metabolism pathways (14). In the *de novo* lipid biosynthesis pathway, sterol regulatory element-binding protein 1 (SREBP1) led to *de novo* lipogenesis and promoted tumor proliferation, which was activated by LMP1 in NPC (15). TINCR, a long noncoding RNA maintained cellular acetyl-CoA synthesis (ACS) in lipogenesis, which was aberrantly upregulated and functioned as an unfavorable prognostic biomarker in NPC, contributing to the carcinogenesis and chemoresistance in NPC (16). These findings suggested that lipid metabolism disorder was considered as a metabolic signature in NPC progression. Thus, targeting lipid metabolism could be an emerging idea in cancer therapy and worth to be further investigated in NPC.

Solute carrier family 27 member 6 (SLC27A6) is a FA transport protein, which regulates long-chain fatty acid (LCFA) uptake and ACS activity (17, 18). In general, the LCFA is abundant in animal tissues, and FAT/CD36, associated with lipid rafts, hand LCFAs directly to SLC27A6 for transport across the plasma membrane (17–19). SLC27A6 was upregulated and considered an invasive biomarker in papillary thyroid carcinoma (20). On the other hand, SLC27A6 was decreased in esophageal squamous cell carcinoma and breast cancer cells (21, 22). Downregulated SLC27A6 inhibited cell proliferation and FA uptake in non-cancerous breast cells but did not affect tumor growth and lipid metabolism in breast cancer (22). These findings suggested that SLC27A6 was involved in tumorigenesis and lipid metabolism.

The present study illustrated the epigenetic inactivation of SLC27A6 in NPC. Re-expression of SLC27A6 significantly inhibited cell proliferation and clonogenicity but promoted tumor migration and invasion both *in vitro* and *in vivo*. Furthermore, we observed an activation of the epithelial-mesenchymal transition (EMT) in a tumor xenograft. Overexpression of SLC27A6 also increased FA uptake, negatively regulated ROS level and positively correlated with cancer stem cell (CSC) markers in NPC cells. Our data presented new insights into the mechanism of SLC27A6 in lipid metabolism and revealed a dual role in NPC progression.

## Materials and Methods

### Bioinformatic Analysis

The mRNA expression and methylation degree of SLC27A6 were performed based on the Gene Expression Omnibus database. Six microarray datasets (GSE12452, GSE13597, GSE39826, GSE40290, GSE53819, GSE64634) were used for gene expression analysis, while a microarray dataset (GSE62336) was used for methylation analysis.

### Human Samples

The NPC primary tumor specimens from 65 newly diagnosed patients were used in this study. Nasopharyngeal epithelia obtained from the 42 donors’ normal nasopharyngeal epithelia (NNE) samples were used as controls. All donors signed informed consent forms. Experienced pathologists confirmed pathological diagnose based on the WHO classification. Among them, 26 primary NPC biopsies and 19 NNE were used for RNA isolation. And, 19 NPC tissues and 14 NNE were used for immunohistochemistry (IHC) staining. Another 20 NPC and nine NNE were used for bisulfite sequencing.

### Cell Lines and Cell Culture

The immortalized epithelial (NP460) cell line was acquired as a kind present from Professor Sai-Wah Tsao (Hong Kong University) (23–25). Cell lines (CNE1, well-differentiated; 5-8F and HONE1, poorly-differentiated) were cultured in DMEM (high glucose) medium (Gibco, Grand Island, NY, USA) added with 10% FBS and 1% antibiotic antimycotic (26–28). While NP460 was cultured in the medium containing a 1:1 ratio mixture of DK-SFM (Gibco, Grand Island, NY, USA) with growth factors and epilife medium, and other components were listed as previously described (29).

### RNA Isolation and Quantitative Real-Time PCR (qRT-PCR)

As previously described, total RNA isolation, first-strand cDNA synthesis, and qRT-PCR were performed (30). The primers were listed in **Supplementary Table S1**. The transcriptional gene expression was performed with SYBR Green Supermix (Qiagen, Hilden, German) by the qRT-PCR System (StepOnePlus, Applied Biosystems, Waltham, MA, USA). The relative transcriptional level of SCL27A6 was normalized to β-actin mRNA expression and calculated using the 2^−ΔΔCt^ method (31).

### Bisulfite Sequencing

The MethylTarget^®^ method (Genesky Biotechnologies Inc., Shanghai, China) was applied to detect the DNA methylation rate. For sodium bisulfite treatment, 400 ng genomic DNA was conducted by using EZ DNA Methylation™-GOLD Kit (Zymo Research, Irvine, CA, USA). The standard protocols were performed as previously described (32). The primers used for SLC27A6 amplification were summarized in **Supplementary Table S1**.

### 5-aza-2’-deoxycytidine (5-aza-dC) Demethylation Treatment

These three cell lines (CNE1, HONE1, 5-8F; 1×10^5^) were seeded into six-well plates and incubated for four days with five µM 5-aza-dC (Sigma-Aldrich, St. Louis, MO, USA). The fresh medium added with 5-aza-dC was changed every 24 h. After incubation for four days, cells were harvested, and mRNA expression was investigated by qRT-PCR.

### Transfection

NPC cell lines (HONE1, 5-8F) were stably transfected with the SLC27A6-containing plasmid or control vector plasmid pCMV6-entry (Origene, Rockville, MD, USA) using Lipofectamine 3000 (Invitrogen, Carlsbad, CA, USA). SLC27A6 ORF cDNA was amplified and subcloned into the pCMV6-entry vector. Stable clones of SLC27A6 (experimental group, SLC27A6-HONE1, SLC27A6-5-8F) or control vector plasmid pCMV6-entry (control group, Ctrl-HONE1, Ctrl-5-8F) were respectively obtained by G418 selection (600, 200 μg/mL) for two weeks. SLC27A6 expression was confirmed by qRT-PCR and western blotting.

### Cell Proliferation Assay

The function of the SLC27A6 gene on cell growth was tested with the Cell Counting Kit-8 (CCK-8) assay (Dojindo, Kumamoto, Japan). Briefly, SLC27A6-HONE1/5-8F and Ctrl-HONE1/5-8F cells (1×10^3^) were seeded into 96-well plates. Subsequently, the cell proliferation assay was tested every 24 h for four days. The OD values were determined at 450 nm.

In addition, SLC27A6-5-8F and Ctrl-5-8F cells were treated with oleic acid (OA) in two concentrations (30 µM and 45 µM). Cells were treated in a medium supplemented with OA for 48 h. After treatment, cells (1×10^3^) were plated in each well of 96-well plates. Similarly, the cell proliferative capacity was measured by the CCK-8 assay as mentioned above.

### Colony Formation Assay

Stably transfected NPC cells (HONE1, 5-8F; 2×10^2^) were seeded into six-well plates. Cells were then cultured for two weeks. Then colonies were washed with PBS, fixed with 70% ethanol, stained by Giemsa staining, and calculated utilizing Quantity One v4.4.0 (Bio-Rad, Hercules, CA, USA). The experiments were repeated twice.

### Wound Healing Assay

Stably transfected NPC cells (5×10^5^) grow into six-well plates with 10% FBS culture media for up to 90% confluence. A sterile pipette tip (1000 μl) was utilized to scratch the monolayer cells. After six hours, wound closure was investigated by an inverted phase microscope (TS100, Nikon, Japan). The experiments were conducted in triplicate.

### Transwell Assay

Cells (2×10^4^) were plated into the upper chambers of BioCoat Migration Chambers (BD, Bedford, MA, USA) without Matrigel for migration assay, while cells (3×10^4^) were plated into the upper chamber of Invasion Chambers coated with Matrigel for invasion assay. After 24 h incubation, non-migrating or non-invading cells were eliminated by using swabs. Cells on the lower membrane surface, which involved the migratory or invasive cells, were fixed with 4% fixative solution, stained with 0.1% crystal violet solution, and photographed.

### *In Vivo* Xenograft Models

As described previously, xenograft models were established (33). Control group cells or experimental group cells (1×10^6^) were implanted into a nude mouse (BALB/c-nu, male, 4-week-old; Vital River Laboratory Animal Technology, Beijing, China) left flank to generate a subcutaneous xenograft model. All mice were fed in a Specific Pathogen Free animal lab and were randomly assigned into the experimental and control group.

### Western Blotting

For western blotting, protein samples were measured according to the standard protocol described previously (34). The following antibodies were used: SLC27A6 (1:1000 dilution, ab84183, Abcam, Hangzhou, China) and GAPDH (1:1000 dilution, HRP-60004, Proteintech, Chicago, IL, USA).

### Immunohistochemical Staining

For IHC analysis, standard methods were applied as previously described (31). Antibodies SLC27A6 (1:100 dilution, ab84183, Abcam, Hangzhou, China), Ki-67 (1:100 dilution, ab15580, Abcam, Hangzhou, China), E-cadherin (1:400 dilution, 3195, Cell signaling technology, Ma, USA), β-catenin (1:100 dilution, 8480, Cell signaling technology, Ma, USA), and Snail (1:50 dilution, 3879, Cell signaling technology, Ma, USA) were used in this study. Two independent investigators performed IHC scores based on staining intensity and staining frequency.

### Flow Cytometric Assessment

For flow cytometry analysis, cells (SLC27A6-HONE1/5-8F and Ctrl-HONE1/5-8F) were collected to determine LDs levels. For lipid peroxide assay, the cells were washed and incubated with DAPI (C0065, Solarbio, Beijing, China) for 0.5 h. Then, the cells were incubated with BODIPY (3932, Invitrogen, Carlsbad, CA, USA) staining solution in the dark for 0.5 h. Cells were washed with a quick rinse using PBS to remove the staining solution. The supernatant was carefully aspirated, and the cell pellets were resuspended in 350 μl 1× PBS. The cell suspension was filtered through a 35 μm membrane into a FACS tube and subjected to flow cytometry.

Moreover, CD44 expression levels in SLC27A6-HONE1/5-8F or Ctrl-HONE1/5-8F cells were measured by flow cytometry. Based on the standard protocol, cells were incubated with CD44 (#12-0441-82, Invitrogen, Carlsbad, CA, USA) and its Rat IgG2b kappa Isotype Control (#12-4031-82, Invitrogen, Carlsbad, CA, USA) in the dark for 0.5 h, respectively.

### Triglyceride (TG) and Total Cholesterol (T-CHO) Detection

TG detection kit (A110-1-1, NJJC, Nanjing, China) and T-CHO detection kit (A111-1-1, NJJC, Nanjing, China) were used to measure TG and T-CHO levels following the manufacturer’s protocols, respectively. The OD value (510 nm) was assessed in a microplate reader (BioTek, Winooski, VT, USA).

### Reactive Oxygen Species (ROS) Assessment

The ROS assay was conducted using a ROS assay kit (S0033S, Beyotime, China). Cells were incubated with a DCFH-DA probe (1:1000 dilution) at 37°C for 0.5 h. Then the fluorescence intensity was detected by a Micro Fluorescence Reader with excitation at 488 nm (BIO-TEK Instruments, Winooski, VT, USA).

### Statistical Analysis

Data was performed with SPSS 26.0 (SPSS, Chicago, IL, USA). The unpaired Student *t*-test was used to compare data between two groups. The Mann-Whitney *U*-test determined statistical differences for IHC. A *P* value < 0.05 was considered as statistically significant (*P < 0.05, **P < 0.01, ***P < 0.001 vs. control group).

## Results

### SLC27A6 Is Downregulated in NPC Primary Tissues and Cells

To investigate the consistency of abnormal mRNA expression of SLC27A6 in NPC, we analyzed six microarray datasets involving 114 NPC and 46 NNE tissues *via* meta-analysis (**Supplementary Table S2**). The results showed that individual dataset had significant heterogeneity (I_2_ = 56.0%, *P* < 0.05) and the pooled Standard Mean Difference (SMD) as -1.67 (95% CI: -2.38, -0.97, **Supplementary Figure S1A**). There was no significant difference in the sensitivity analysis (**Supplementary Figure S1B**). In addition, there was no publication bias in the study (Egger’s regression test: *P=0.303*, **Supplementary Figure S1C**).

**Figure 1:**
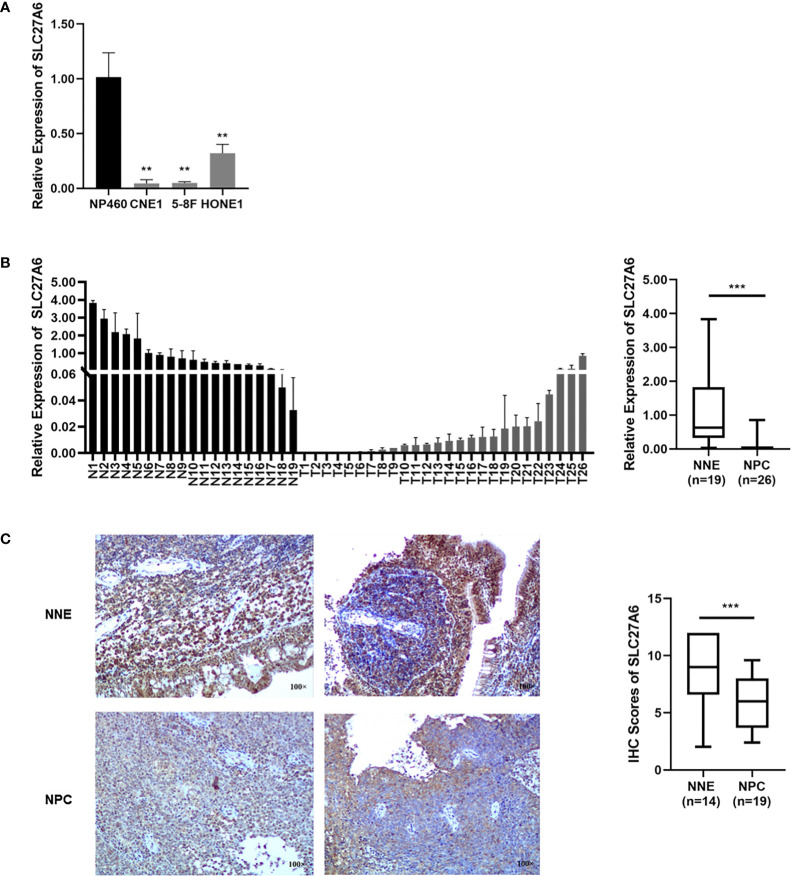
The SLC27A6 expression is decreased in NPC. **(A)** SLC27A6 mRNA expression was tested by qRT-PCR in NPC cell lines (CNE1, 5-8F, and HONE1) and an immortalized epithelial (NP460) cell line. **(B)** SLC27A6 mRNA expression in primary NPC tissues (n=26) and NNE (n=19) specimens. **(C)** IHC staining of SLC27A6 in primary NPC tissues (n=19) and NNE (n=14) specimens. Magnifications ×100. **P < 0.01 and ***P < 0.001.

We then assessed the SLC27A6 transcriptional level in NPC cell lines by qRT-PCR. SLC27A6 expression was remarkably decreased in NPC cells (CNE1, 5-8F, HONE1) compared with NP460 (**Figure 1A**). In addition, the SLC27A6 mRNA expression was also downregulated in NPC primary tissues (n=26) than in NNE (n=19) (**Figure 1B**). The expression of SLC27A6 protein was more robust in the cytosol and membrane of NNE (n=14) samples while weaker in NPC (n=19) samples. The SLC27A6 protein expression was markedly lower in NPC patients (**Figure 1C**). Our results were consistent with the meta-analysis, indicating low expression of SLC27A6 in NPC.

### SLC27A6 Is Inactivated *via* DNA Promoter Hypermethylation

**Figure 2:**
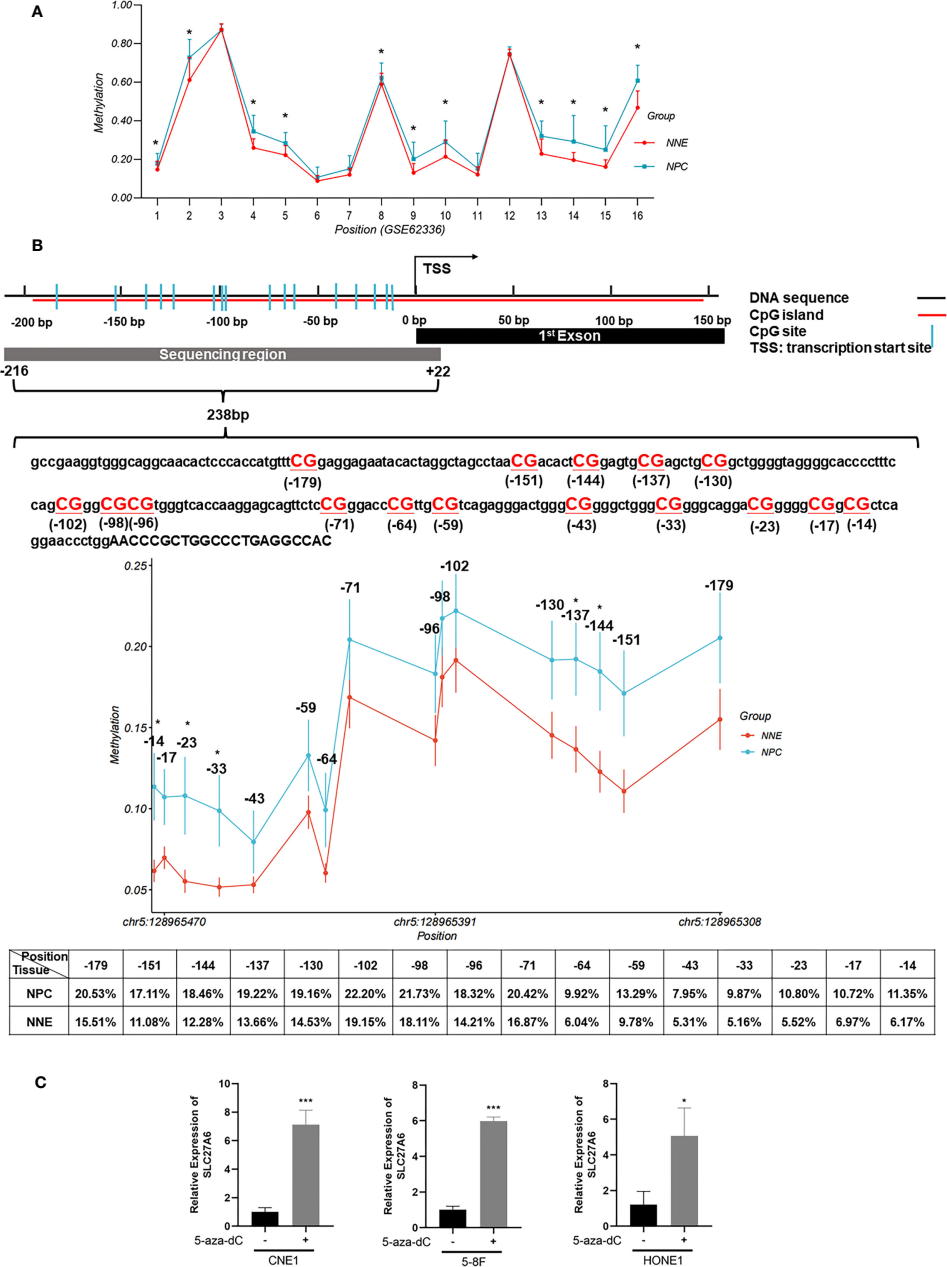
DNA promoter hypermethylation of SLC27A6 in NPC and NNE tissue. **(A)** The promoter region of SLC27A6 included 16 CpG sites, and the average methylation ratio was analyzed *via* DNA methylation microarray data (GSE62336; 25NPC, 25NNE). **(B)** The extent of CpG methylation in 238 bp region within the SLC27A6 promoter was analyzed by the bisulfite gene sequencing (NPC biopsies, n=20; NNE biopsies, n=9). **(C)** SLC27A6 mRNA expression in three NPC cell lines treated or untreated with demethylation reagent. *P < 0.05; ***P < 0.001.

DNA promoter methylation is the most well-characterized epigenetic in NPC, involving enzymes belonging to the DNA methyltransferase (DNMT) family (35). To investigate whether SLC27A6 was a low expression by promoter hypermethylation, we explored the SLC27A6 promoter using EMBOSS (https://www.ebi.ac.uk/Tools/emboss/). A CpG island with a length of 333bp (-190 ~ +142 bp from the transcription starting site, TSS) was seen in the promoter region of SLC27A6. Next, we explored promoter methylation degree through the methylation microarray dataset (GSE62336). This promoter region includes 16 CpG sites, and we found a higher average methylation rate in CpG sites (11/16) in NPC tissues compared with normal tissues (**Figure 2A**). These results indicated that the promoter methylation modification of SLC27A6 was significantly stronger in NPC than in NNE samples.

We sought to explore the promoter methylation ratio of SLC27A6 in NPC (n=20) and NNE (n=9) tissues. The bisulfite gene sequencing was performed to detect 16 CpG sites from the TSS (-216 ~ +22 bp) of the SLC27A6 promoter. **Figure 2B** showed the schematic model and DNA sequence of SLC27A6 promoter region analyzed by bisulfite gene sequencing. In addition, we listed the individual CpG sites methylation rate between NPC and NNE tissues in **Supplementary Table S3**. Also, a higher promoter methylation rate of SLC27A6 was observed in all CpG sites in the NPC than in NNE tissues. Among them, five CpG sites, including chr5: 128965343, chr5: 128965350, chr5: 128965454, chr5: 128965464, chr5: 128965473, showed statistical differences.

To further explore the inactivation mechanism of SLC27A6 expression, three NPC cell lines were treated with the demethylation reagent 5-aza-dC. As a DNMT inhibitor, 5-aza-dC activates the methylation deactivation of gene methylation. When compared to the control (DMSO) cells, the mRNA expression level of SLC27A6 was significantly upregulated by seven-fold (CNE1), six-fold (5-8F), five-fold (HONE1), respectively. CNE1 treated with 5-aza-dC showed maximum recovery. The results showed that the expression of SLC27A6 mRNA expression was dramatically restored after demethylation treatment (**Figure 2C**). It estimated that DNA promoter CpG island hypermethylation might be one of the reasons leading to the downregulation of SLC27A6.

### SLC27A6 Inhibits Cell Proliferation and Colony Formation *In Vitro*

**Figure 3:**
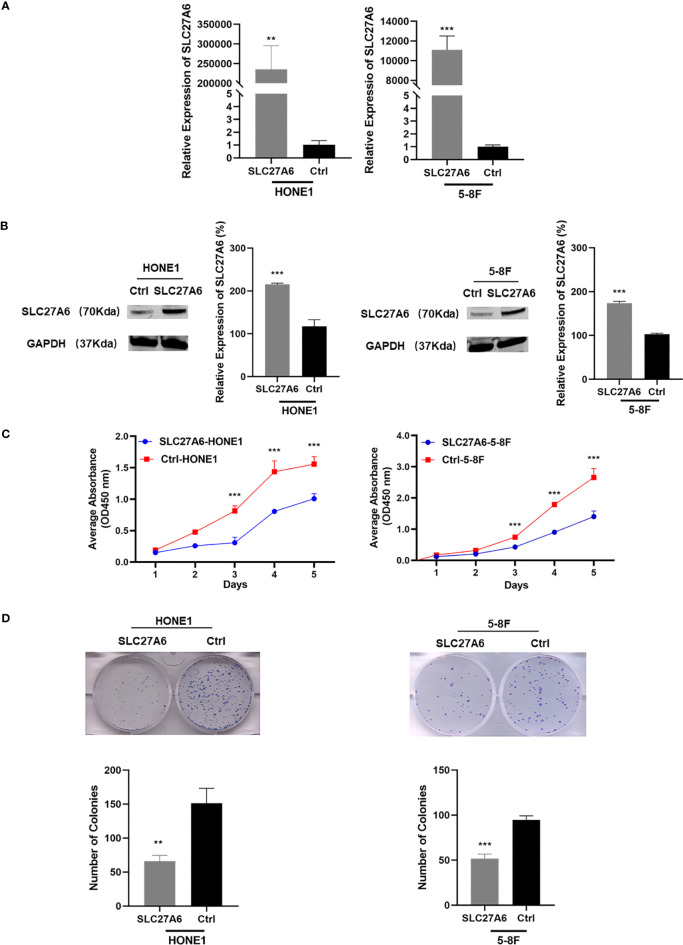
Overexpression of SLC27A6 suppresses cell proliferation. **(A, B)** SLC27A6 mRNA expression in stably transfected NPC cells (HONE1, 5-8F) was confirmed by qRT-PCR **(A)** and western blotting **(B)**. NPC cells were stably transfected with control vector plasmid pCMV6-entry as the control group (control group, Ctrl-HONE1, Ctrl-5-8F). **(C)** Cell proliferation in stably transfected NPC cells (HONE1, 5-8F) was measured by CCK-8 assay (OD=450 nm). **(D)** Colony formation assay tested in stably transfected NPC cells (HONE1, 5-8F). **P < 0.01 and ***P < 0.001.

To investigate the potential roles of SLC27A6 on the malignant phenotype of NPC cells, we stably expressed SLC27A6 in two NPC cell lines (HONE1, 5-8F). Ectopic overexpression of SLC27A6 was checked by qRT-PCR and western blotting (**Figures 3A, B**). We found that overexpression of SLC27A6 substantially suppressed NPC cell proliferation (**Figure 3C**). In addition, SLC27A6 also considerably reduced NPC cell colony formation (**Figure 3D**) compared with the control group. The clone formation rate of SLC27A6 was only 44% compared with HONE1 control cells and 55% compared with 5-8F control cells, respectively.

### SLC27A6 Suppresses Tumorigenesis *In Vivo*

**Figure 4:**
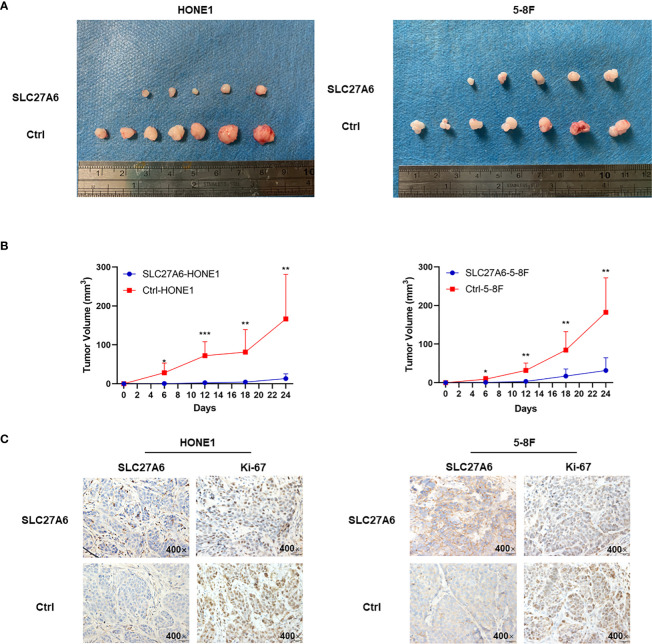
Overexpression of SLC27A6 reduces NPC cells tumorigenesis *in vivo*. **(A)** Xenograft model in nude mice from injected SLC27A6-HONE1/5-8F or Ctrl-HONE1/5-8F cells (n=7). **(B)** The tumor growth of re-expression of SLC27A6 in SLC27A6-HONE1/5-8F and Ctrl-HONE1/5-8F cells-derived subcutaneous. **(C)** IHC staining of SCL27A6 and Ki-67 in xenografts. Magnifications ×400. *P < 0.05; **P < 0.01; ***P < 0.001.

As SLC27A6 overexpression displayed inhibition ability of tumor growth *in vitro*, we investigated whether SLC27A6 had similar effects *in vivo*. We established xenograft tumor in nude mice using two stably transfected cells (HONE1, 5-8F). After the NPC cells were inoculated into nude mice, tumorigenicity was 100% (7/7) in the control group, but only 71.4% (5/7) in the SLC27A6 group in both HONE1 and 5-8F (**Figure 4A**). In addition, the mice injected with SLC27A6-HONE1/5-8F cells showed slower growth and smaller tumor size than the control group (**Figure 4B**). These results demonstrated that the xenograft tumor growth was dramatically inhibited by SLC27A6 overexpression *in vivo*. Moreover, ectopic overexpression of SLC27A6 significantly suppressed Ki-67 protein expression (**Figure 4C**), a marker for cell proliferation. Together, these results indicated that SLC27A6 repressed tumorigenicity *in vivo*.

### SLC27A6 Enhances Cell Metastasis in NPC Cells

**Figure 5:**
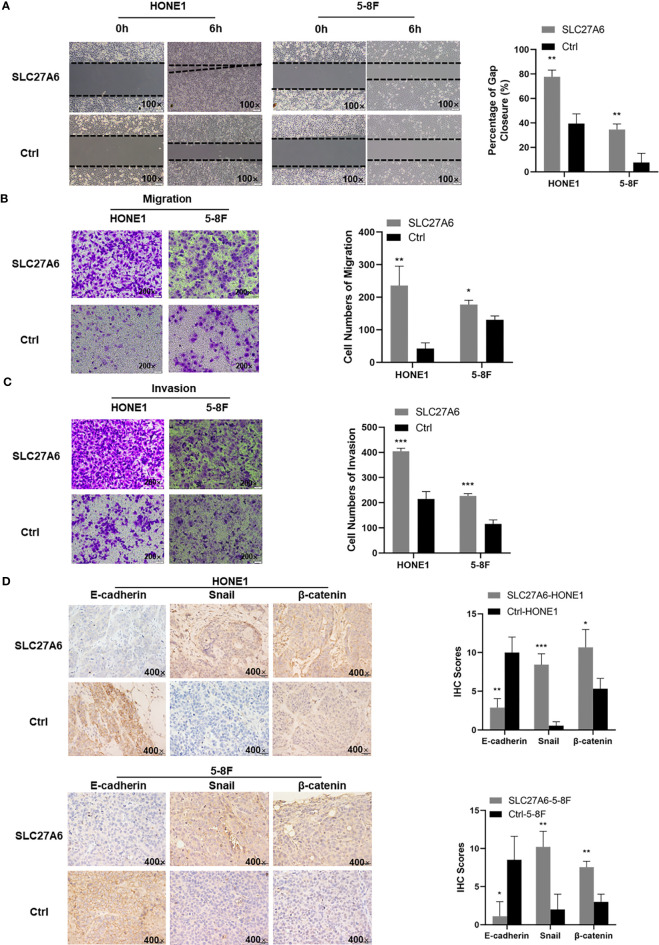
Overexpression of SLC27A6 facilitates the migration, invasion and EMT in NPC cells. **(A)** Detection of migration in stably transfected NPC cells (HONE1, 5-8F) using a wound-healing assay. Magnification ×100. **(B)** Migration differences in stably transfected NPC cells (HONE1, 5-8F) by transwell assay. **(C)** Detection in invasion of stably transfected NPC cells (HONE1, 5-8F) using transwell assay (gel-coated). Magnification ×200. **(D)** IHC staining of the EMT signals (E-cadherin, Snail and β-catenin) in xenograft tumor. Magnifications ×400. *P < 0.05; **P < 0.01; ***P < 0.001.

The ability of metastasis in cancer is essential in NPC. We next evaluated the effect of SLC27A6 in the context of metastasis *in vitro*. To our surprise, results showed that overexpression of SLC27A6 promoted the wound closure rate compared with their control cell. In HONE1 stable cells, the percentage of gap closure was 77% in SLC27A6 overexpression cells, while only 40% in HONE1 control cells. Similar to HONE1, the percentage of gap closure was 35% in SLC27A6 overexpression cells while only 8% in 5-8F control cells (**Figure 5A**). Consistently, overexpression of SLC27A6 also boosted migration through migration chambers in these stably transfected cells (**Figure 5B**). We next examined invasive properties of SLC27A6 in NPC cells. Interestingly, HONE1 and 5-8F cells stably expressing SLC27A6 showed stronger invasion ability through Matrigel-coated invasion chambers than control cells (**Figure 5C**). EMT is known to be closely linked to metastasis. Next, we performed IHC staining to examine the effect of SLC27A6 on molecular markers of EMT in a xenograft mouse model. In particular, ectopic expression of SLC27A6 markedly suppressed expression of epithelial marker (E-cadherin), but increased expression of EMT transcription factors (Snail) and enhanced expression of β-catenin signaling in xenograft tumor (**Figure 5D**). Collectively, our data showed that SLC27A6 enhanced the ability of metastasis in NPC cells both *in vitro* and *in vivo*.

### SLC27A6 Promotes FA Uptake and Downregulates ROS in NPC Cells

**Figure 6:**
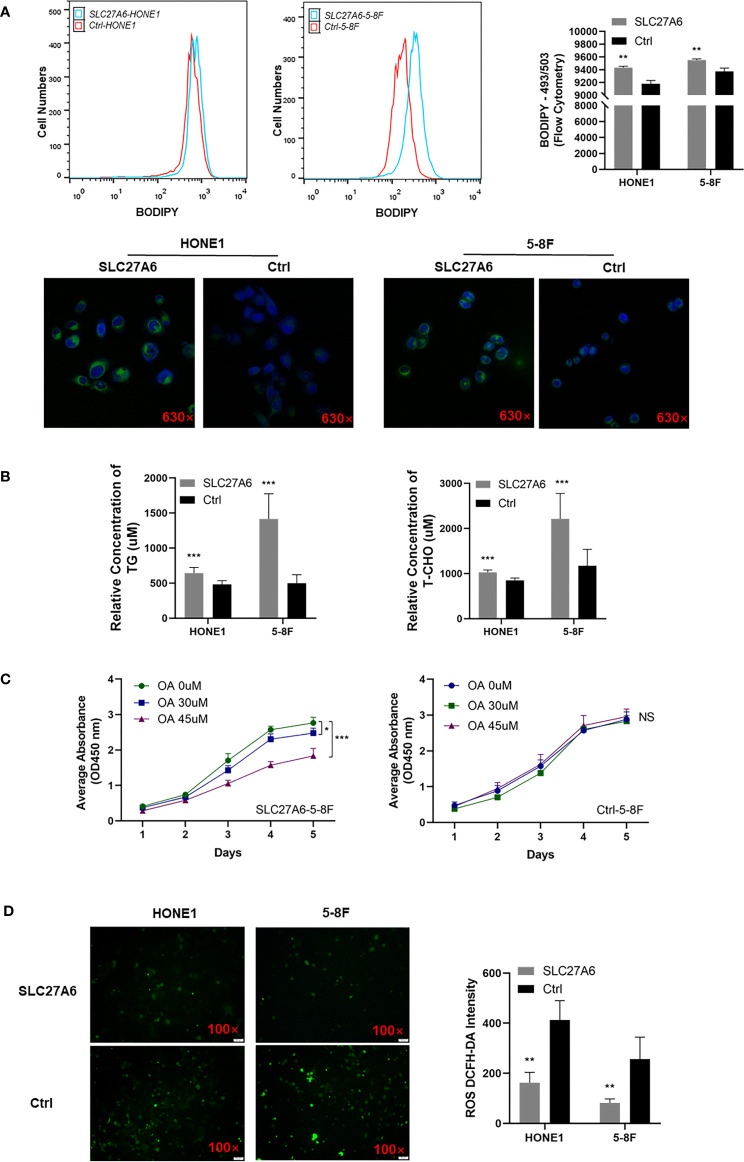
Overexpression of SLC27A6 induces lipid accumulation inhibiting cell proliferation in NPC cell lines. **(A)** LDs incubated with BODIPY (493/503) (green) and nuclei with DAPI (blue) in NPC cell lines. Magnification ×630. **(B)** The relative concentration of TG and T-CHO in SLC27A6-HONE1 and SLC27A6-5-8F cells contrasted to control cells. **(C)** Cell proliferation with OA treatment in SLC27A6-5-8F cell (left panel) and control group (right panel) measured by CCK-8 assay (OD=450 nm). **(D)** ROS detection in SLC27A6-HONE1/5-8F and Ctrl-SLC27A6/5-8F, followed by intensity analysis. Magnification ×100. NS, no significance > 0.05; *P < 0.05; **P < 0.01; ***P < 0.001.

Considering the role of SLC27A6 involved in LCFA uptake and ACS, including β-oxidation and TG synthesis, we investigated whether overexpression of SLC27A6 increased the lipid content of NPC cells. We stained a lipid-specific fluorescent dye (BODIPY) in stably transfected SLC27A6 NPC cells and the control group. Our study revealed a link between lipid metabolism and SLC27A6 expression in NPC. We found that experimental groups contained more LDs than control groups by flow cytometry. Then microscopy revealed NPC cells stained more robust intracellular LDs in the cytoplasm (**Figure 6A**). We further explored TGs and cholesterol by flow cytometry, which are primarily composed of LDs. As expected, SLC27A6 expression cells contained more TG and T-CHO intracellularly (**Figure 6B**). These results might imply that the high expression of lipid metabolism was related to the overexpression of SLC27A6.

To investigate whether intracellular lipid accumulation impaired tumor growth, we treated NPC cells with OA. Interestingly, OA inhibited cell proliferation in SLC27A6 overexpression cells *in vitro* (**Figure 6C**, left panel), and the result was positively correlated with the concentration of OA. However, OA had not affected the control group in NPC cells (**Figure 6C**, right panel). Thus, our data suggested that SLC27A6 regulated lipid metabolism in response to the lipid-rich environment in NPC.

Considering that LCFA regulated intracellular production of ROS, we then analyzed the ROS level in SLC27A6 stably transfected NPC cells. The results showed that overexpression of SLC27A6 decreased ROS levels in stably transfected cells (**Figure 6D**). This indicated that SLC27A6 mediated lipid uptake and negatively regulated ROS levels.

### SLC27A6 Is Positively Associated With CSCs in NPC Cells

**Figure 7:**
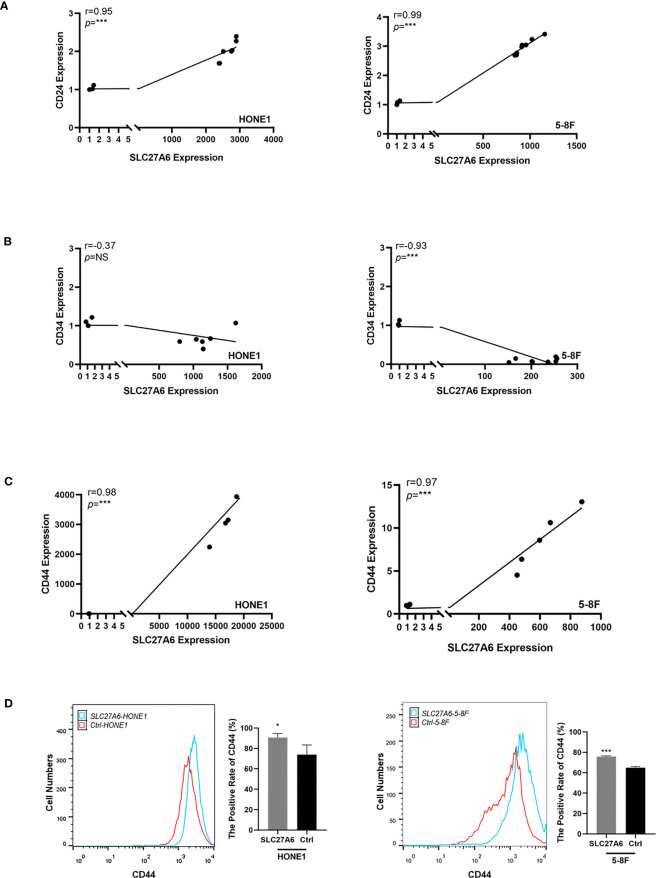
SLC27A6 expression positively correlates with CD24 and CD44 expression level in NPC cells. **(A)** Positive correlation between the mRNA expression of SLC27A6 and CD24 in NPC cells (HONE1, R=0.95; 5-8F, R=0.99) by qRT-PCR. **(B)** No significant difference was shown between the SLC27A6 and CD34 mRNA expression in HONE1 (left panel), while a negative correlation was seen in 5-8F (right panel). **(C)** Positive correlation between the mRNA expression of SLC27A6 and CD44 in NPC cells (HONE1, R=0.98; 5-8F, R=0.97) by qRT-PCR. **(D)** The CD44 expression levels in HONE1 (left panel) and 5-8F (right panel) stably transfected cells were measured by flow cytometry. NS, no significance > 0.05; *P < 0.05; **P < 0.01; ***P < 0.001.

CSCs actively promote tumor metastasis by generating high cell turnover. To explore whether SLC27A6 was associated with CSCs, we analyzed the correlation between SLC27A6 and CSC markers in NPC cells by qRT-PCR, including CD24, CD34, and CD44 expression. As shown in **Figures 7A** **, C**, a positive correlation between SLC27A6 and CD24, CD44 expression was observed. However, there was no significant difference between SLC27A6 and CD34 in the HONE1 cell (**Figure 7B**, left panel), and a negative correlation between SLC27A6 and CD34 in 5-8F cell (**Figure 7B**, right panel). Furthermore, we evaluated the CD44 expression in stably transfected cells by flow cytometry. CD44 expressed at higher levels in the SLC27A6-HONE1/5-8F group than in the control group (**Figure 7D**). To some extent, we speculated SLC27A6 promoted NPC metastasis *via* increasing CD24 and CD44 positive tumor stem cells.

## Discussion

Epigenetic alterations, including differential modification of DNA, RNA, proteins, miRNA, have been widely observed in the progression of NPC (36, 37), leading to the inactivation of a series of TSGs. Among them, DNA promoter CpG island hypermethylation was the most frequently reported mechanism in NPC (38, 39), such as RASSF1A, RASSF2A, CDKN2A, ADAMTS18 (5, 40–43), which contributed to the early pathogenesis, even earlier than EBV infection (38, 44). SLC27A6 promoter hypermethylation was firstly reported in colorectal cancer (45). Here, our study demonstrated that SLC27A6 was frequently inactivated in primary NPC tissues and cell lines by CpG island hypermethylation of the DNA promoter.

In this study, a complex view of the aspect of tumorigenesis modulated by SLC27A6 has been addressed. We found that overexpression of SLC27A6 significantly inhibited tumorigenesis *in vitro* but promoted wound closure rate, migration, and invasion in NPC cells alternatively. We further confirmed that SLC27A6 overexpression was significantly suppressed the xenograft tumor growth and tumor size *in vivo*. EMT is a cellular process characterized by loss of epithelial properties and acquisition of mesenchymal phenotype. It is associated with tumor migration, invasion, metastasis, and tumor stemness (46, 47), and poor prognosis in multiple cancers (48, 49), including NPC (50). Here, we showed that overexpression of SLC27A6 induced EMT in NPC cells by interfering with E-cadherin expression, upregulating with EMT transcription factors (Snail), and activating β-catenin signaling, could result in tumor metastasis *in vivo*. Our data suggested that SLC27A6 played both anti-tumorigenic and pro-metastatic roles in NPC. Unlike the classical TSGs silenced by DNA promoter CpG island hypermethylation in NPC, overexpressing of SLC27A6 played a dual role in NPC cells. SLC27A6 was silenced in primary NPC tissues and cell lines by DNA promoter CpG island hypermethylation. In addition, the demethylation treatment restored its expression in NPC cell lines. As DNA promoter CpG island hypermethylation is a critical mechanism for TSGs in NPC (37, 38), we estimated that epigenetic silence of SLC27A6 was a part of the mechanism in NPC process. In addition, re-expression of SLC27A6 had an anti-tumorigenic role in repressing proliferation in NPC cells and inhibited tumor growth in xenografts. However, SLC27A6 overexpression exhibited pro-metastatic functions by promoting tumor migration and invasion *in vitro* and facilitating EMT of metastatic lesions *in vivo*.

Energy metabolic reprogramming has been suggested as an essential feature of cancer progression (8). Alterations in lipid metabolism were essential for cancer cell proliferation, motility, and differentiation (12, 13). Increasingly evidence supported that dysregulation of metabolism was observed in NPC oncogenesis, including enhancing aerobic glycolysis, LDs accumulation, and iron overload (14, 33, 51). LDs accumulation was essential for providing energy to cancer cells, inducing cancer cell proliferation (12). Previously, we observed lipid accumulation in primary NPC compared with NNE tissues and cells (14, 52). Kwok-Wai Lo et al. reported that SREBP1 mediated lipid synthesis and contributed to cell growth. In addition, LMP1 induced SREBP1-mediated lipogenesis *via* targeting FASN (15). Moreover, increased lipid turnover and FA oxidation activation were observed in radiation-resistant NPC cells. And CPT1A induced FA trafficking and radiation resistance (53).

On the other hand, lipid metabolism reprogramming can promote cancer metastasis (54). For instance, FASN expression facilitated peritoneal metastasis by mediating EMT in ovarian cancer (55). In NPC, knockdown ATGL showed LDs accumulation and increased migration in LMP2A positive NPC cells (14). In line with our results, SLC27A6-mediated lipid accumulation increased migration and invasion ability in NPC. These results suggested that changes in fat and lipid metabolism were observed in NPC, which involved tumorigenesis and cancer development. SLC27A6 expression was highly increased in enzalutamide-resistant prostate cancer (56). SLC27A6 was a long-chain transport protein involved in LCFAs transport across the plasma membrane (17). Upregulation of SLC27A6 restored lipids and fats levels, essential for maintaining cell proliferation (22, 56). We confirmed that SLC27A6 overexpression significantly enhanced either FAs, TG, and T-CHO in NPC cells. However, increasing LDs suppressed tumorigenesis both *in vitro* and *in vivo*. FA, TG, and T-CHO are the main components of LDs. Within cells, there are some fates for FAs, such as membrane lipid synthesis, storage, or oxidization to carbon dioxide (12). Besides, when the lipid homeostasis blocks cells, the lipid accumulation may cause lipotoxicity, leading to cell damage (57). Lipid overload increased the levels of FAs in cells and was associated with elevated β-oxidation, lipid peroxidation, mitochondrial damage, ER stress, impaired insulin signaling, increasing inflammatory mediators, and cell death, which may account for parts of reasons why SLC27A6-mediated lipid overload inhibited tumor growth in NPC.

In mitochondria, the respiratory chain is a major source of ROS (58), and FA exhibits a dual effect on ROS production. On the one hand, lipid accumulation increases ROS generation in forward electron transport; on the other hand, due to the protonophoric action in the inner mitochondrial membrane, FA inhibits ROS production in reverse electron transport (59). An elevated ROS level contributed to rapid cell growth and metastasis in tumor cells (60–62). In this study, SLC27A6 overexpression negatively regulated ROS levels in NPC cells. We found that SLC27A6 promoted lipid accumulation but eliminated the ROS level. Thus, it was reasonable to suppose that SLC27A6 enhanced lipid storage and provided insufficient FAs for tumor growth, and negatively regulated the ROS pathway.

Interestingly, a lower ROS level was associated with CSCs maintaining, which is beneficial for cell survival (63, 64). In addition, alteration of lipid metabolism also facilitated cancer metastasis through regulating CSCs (54). FASN promoted maintaining CSC stemness and was connected with cell proliferation and invasion ability in glioblastoma (65). Lipid desaturation acted as a metabolic marker and promoted CSC phenotype in breast cancer cells (66). Lipid rafts, enriched with sphingolipids and cholesterol, regulated the interaction between CD44 and hyaluronan, mediating cancer cell migration (67). CSC was one of the major factors resulting in metastasis in multiple tumors (68, 69). Mounting evidence suggested that CD44 and CD24 were surface CSC markers involved in cell adhesion and migration in NPC (34, 70–72). The positively expressed rate of CD44 was about 52.5% in 5-8F (72). We investigated the positive relationship between SLC27A6 and CSC markers (CD24, CD44) in stably transfected cells by qRT-PCR. Furthermore, we found that the CD44 expression level in HONE1/5-8F stably transfected cells was higher than in the control group by flow cytometry. The direct link between SLC27A6 expression and CSC markers indicated that SLC27A6 was associated with cancer metastasis ability. Thus, we supposed that overexpression of SLC27A6 upregulated the lipid intake, further increasing lipid accumulation in NPC cells. The excessive LDs could upregulate CD24, CD44 expression and promote maintaining NPC CSC stemness, which could be a possible mechanism explaining why SLC27A6 overexpression promoted cancer metastasis.

## Conclusion

**Figure 8:**
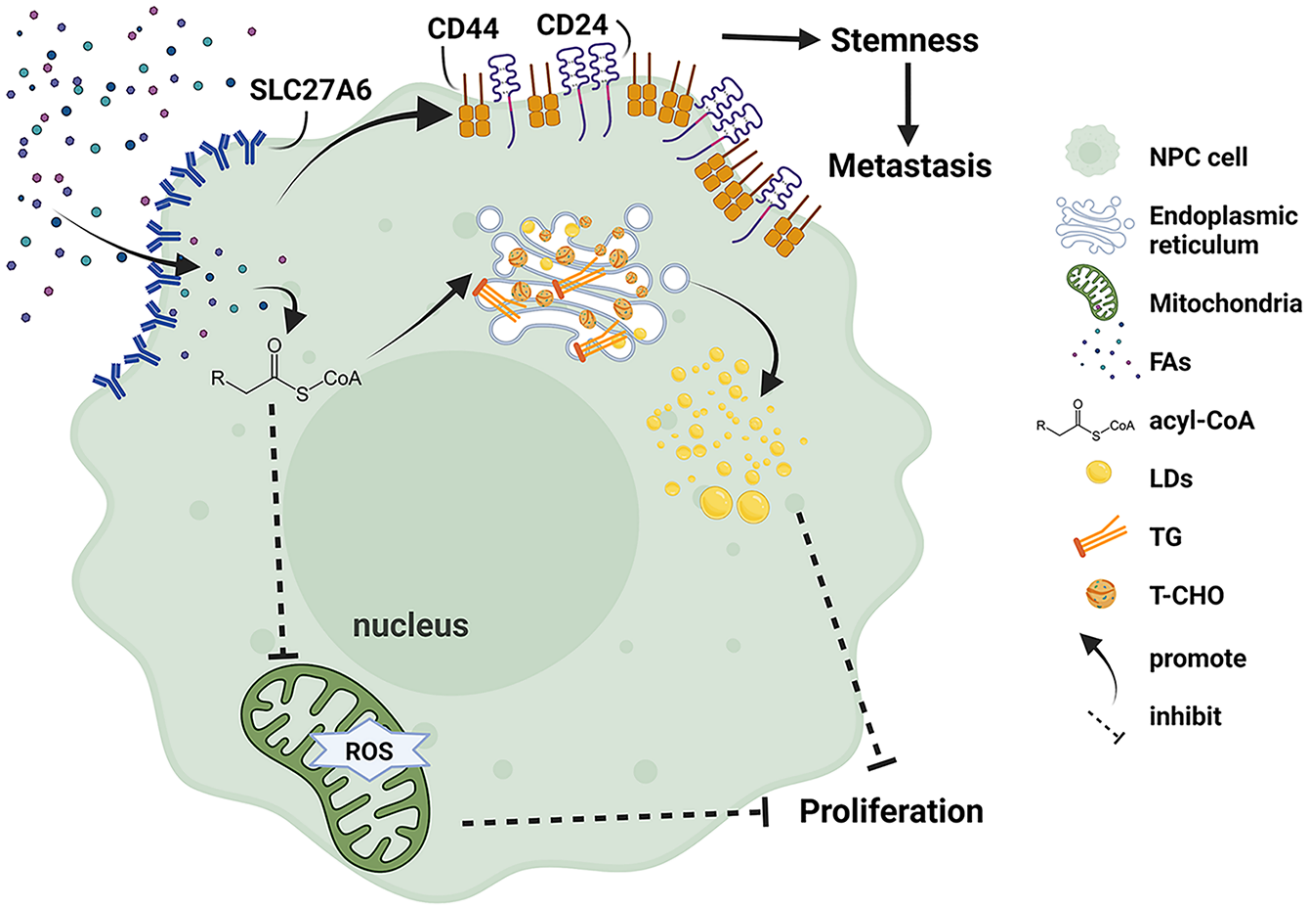
Schematic model illustrating the function of SLC27A6 regulates lipid metabolism in NPC. Re-expression of SLC27A6 increases free FAs uptake of NPC cells. After free FAs are absorbed, they bind to coenzyme A to inform acyl-CoA and convert to TG and T-CHO in the endoplasmic reticulum. In addition, TG and T-CHO are composed of LDs in the cytoplasm. LD storage within cells inhibited tumorigenesis *in vitro* and *in vivo*. However SLC27A6 decreases the level of ROS produced in mitochondria. On the other hand, excessive FAs upregulate the expression of CSC markers (CD24, CD44), maintaining the stemness of NPC CSC and contributing to metastasis.

In conclusion, we elucidated the dual role of SLC27A6 in NPC progression (**Figure 8**). SLC27A6 was silenced by DNA promoter CpG island hypermethylation in NPC. SLC27A6 overexpression repressed cell proliferation and colony formation *in vitro* and inhibited tumor growth *in vivo*. SLC27A6 exerted its proliferation-suppressive function *via* enhancing lipid storage in NPC cells. Nevertheless, SLC27A6 facilitated metastasis through increasing LDs in cells, negatively regulated ROS levels, promoted EMT, and strengthened CSC properties of NPC. The new findings provided a complex insight into SLC27A6 regulating NPC development and progression, which involved lipid metabolism in clinical cancer therapy.

## Data Availability Statement

The datasets presented in this study can be found in online repositories. The names of the repository/repositories and accession number(s) can be found in the article/**Supplementary Material**. The cDNA microarray data comparing the transcriptional level of SLC27A6 in NPC samples and NNE samples were acquired from the GEO database, with search terms as follow: (nasopharyngeal OR nasopharynx) AND (cancer OR carcinoma OR adenocarcinoma OR tumor OR tumor OR malignancy/ malignant* OR neoplasm* OR oncology*). The criteria of inclusion were (1): gene expression data were extracted from homo sapiens (2); samples were all obtained from malignant tissues or non-cancerous NPC tissues (3); both healthy and NPC groups were comprised of at least three cases (4); patients involved did not receive treatment. The STATA 12 software was used for meta-analysis.

## Ethics Statement

The studies involving human participants were reviewed and approved by 2016-KY-050. The patients/participants provided their written informed consent to participate in this study. The animal study was reviewed and approved by 2016-KY-050. Written informed consent was obtained from the owners for the participation of their animals in this study.

## Author Contributions

GWH and WLZ conceived the ideas and designed the research. XMZ and YPY performed experiments. BL, PL, YYH, QZ, and YFW contributed to data analysis. WLZ and XMZ wrote the manuscript. XX, XYZ, YXM, GWH, and ZZ discussed the findings, critically reviewed the manuscript, and supervised experiments. All authors contributed to the article and approved the submitted version.

## Funding

This work was supported by the National Natural Science Foundation of China (81960490, 81760489, 82060511), the Youth Program of Guangxi Natural Science Foundation of China (2018GXNSFBA281158, 2018GXNSFBA281028), the High-level Talent Introduction Plan of the First Affiliated Hospital of Guangxi Medical University (the fifth level), and the fund of Key Laboratory of Early Prevention and Treatment for Regional High Frequency Tumor (Guangxi Medical University), Ministry of Education (GKE-ZZ 202011).

## Conflict of Interest

The authors declare that the research was conducted in the absence of any commercial or financial relationships that could be construed as a potential conflict of interest.

## Publisher’s Note

All claims expressed in this article are solely those of the authors and do not necessarily represent those of their affiliated organizations, or those of the publisher, the editors and the reviewers. Any product that may be evaluated in this article, or claim that may be made by its manufacturer, is not guaranteed or endorsed by the publisher.

## Abbreviation

ACS, acetyl-CoA synthesis; CSC, cancer stem cell; CCK-8, Cell Counting Kit-8; DNMT, DNA methyltransferase; EBV, Epstein-Barr virus; EMT, epithelial-mesenchymal transition; FASN, fatty acid synthase; FAs, Fatty acids; IHC, immunohistochemistry; LCFA, long-chain fatty acid; 5-aza-dC, 5-aza-2’-deoxycytidine; LDs, lipid droplets; NPC, nasopharyngeal carcinoma; NNE, normal nasopharyngeal epithelia; OA, oleic acid; qRT-PCR, quantitative Real-time PCR; ROS, reactive oxygen species; SLC27A6, solute carrier family 27 member 6; SREBP1, sterol regulatory element-binding protein 1; SMD, Standard Mean Difference; TSGs, tumor suppressor genes; TG, triglyceride; T-CHO, total cholesterol.
