# Supplementary material for: Downregulation of SLC27A6 by DNA Hypermethylation Promotes Proliferation but Suppresses Metastasis of Nasopharyngeal Carcinoma Through Modulating Lipid Metabolism
Source: Front Oncol. 2022 Jan 3;11:780410. doi: 10.3389/fonc.2021.780410 (PMC8761909; doi:10.3389/fonc.2021.780410)
Supplement: Supplementary file 1 [file Table_1.docx]

**TABLE S1.** List of primer sequences used in the study.

| **Primers** | **Sequences** | **Note** |
| --- | --- | --- |
| **SLC27A6** | Forward 5’- GAGTTGGGTGCCACTTGTGT-3’ | qRT-PCR |
|  | Reverse 5’- CCAAACGCACCTTATGATCC-3’ |  |
| **CD24** | Forward 5’-GCTCCTACCCACGCAGATTT-3’ | qRT-PCR |
|  | Reverse 5’-GAGACCACGAAGAGACTGGC-3’ |  |
| **CD34** | Forward 5’-ATTGCACTGGTCACCTCGG-3’ | qRT-PCR |
|  | Reverse 5’-TGCGGCGATTCATCAGGAAAT-3’ |  |
| **CD44** | Forward 5’- CCTCACATCCAACACCTCCC-3’ | qRT-PCR |
|  | Reverse 5’- TGTCCCTGTTGTCGAATGGG-3’ |  |
| **β-actin** | Forward 5’- CTTCGCGGGCGACGAT-3’ | qRT-PCR |
|  | Reverse 5’- CCACATAGGAATCCTTCTGACC-3’ |  |
| **SLC27A6** | Forward 5’-GTGGTTTTAGGGTTAGYGGGTTTTAG-3’ | bisulfite sequencing |
|  | Reverse 5’-AACCRAAAATAAACAAACAACACTCC-3’ |  |
