# Supplementary material for: Downregulation of SLC27A6 by DNA Hypermethylation Promotes Proliferation but Suppresses Metastasis of Nasopharyngeal Carcinoma Through Modulating Lipid Metabolism
Source: Front Oncol. 2022 Jan 3;11:780410. doi: 10.3389/fonc.2021.780410 (PMC8761909; doi:10.3389/fonc.2021.780410)
Supplement: Supplementary file 2 [file Table_2.docx]

| **Datasets** | **Year** | **Country** | **SLC27A6** | | | |
| --- | --- | --- | --- | --- | --- | --- |
|  |  |  | **NPC** | | **NNE** | |
|  |  |  | **n** | **Mean±SD** | **n** | **Mean±SD** |
| **GSE12452** | 2008 | USA | 31 | 5.5898±1.0083 | 10 | 7.099±0.9902 |
| **GSE13597** | 2008 | UK | 25 | 1.2677±0.3686 | 3 | 1.557±0.2915 |
| **GSE39826** | 2012 | UK | 3 | 3.5587±0.2124 | 3 | 5.5231±0.0406 |
| **GSE40290** | 2012 | China | 25 | 3.2551±1.879 | 8 | 6.2345±1.3769 |
| **GSE53819** | 2014 | China | 18 | 6.4859±1.4415 | 18 | 9.6832±1.1223 |
| **GSE64634** | 2015 | China | 12 | 3.8169±1.0978 | 4 | 5.2898±1.3212 |

**TABLE S2**. Detailed information on GEO database used for meta-analysis.
