# Supplementary material for: Downregulation of SLC27A6 by DNA Hypermethylation Promotes Proliferation but Suppresses Metastasis of Nasopharyngeal Carcinoma Through Modulating Lipid Metabolism
Source: Front Oncol. 2022 Jan 3;11:780410. doi: 10.3389/fonc.2021.780410 (PMC8761909; doi:10.3389/fonc.2021.780410)
Supplement: Supplementary file 3 [file Table_3.docx]

**TABLE S3.** The DNA methylation rate of SLC27A6 in individual CpG sites.

| **Sites**  **(Distance to TSS)** | **Genome**  **Position** | **Tissue** | |
| --- | --- | --- | --- |
|  |  | **NPC (n=20)** | **NNE (n=9)** |
|  |  | **Mean±SD (**%**)** | **Mean±SD (**%**)** |
| **-14** | chr5: 128965473 | 11.35±9.23 | 6.17±2.00 |
| **-17** | chr5: 128965470 | 10.72±7.60 | 6.97±2.04 |
| **-23** | chr5: 128965464 | 10.80±1.63 | 5.52±2.09 |
| **-33** | chr5: 128965454 | 9.87±9.78 | 5.16±1.72 |
| **-43** | chr5: 128965444 | 7.95±8.59 | 5.31±1.50 |
| **-59** | chr5: 128965428 | 13.29±9.80 | 9.78±3.04 |
| **-64** | chr5: 128965423 | 9.92±10.17 | 6.04±1.75 |
| **-71** | chr5: 128965416 | 20.42±11.09 | 16.87±5.71 |
| **-96** | chr5: 128965391 | 18.32±10.77 | 14.21±4.68 |
| **-98** | chr5: 128965389 | 21.73±10.35 | 18.11±5.49 |
| **-102** | chr5: 128965385 | 22.20±10.08 | 19.15±5.94 |
| **-130** | chr5: 128965357 | 19.16±10.74 | 14.53±4.29 |
| **-137** | chr5: 128965350 | 19.22±9.99 | 13.66±4.24 |
| **-144** | chr5: 128965343 | 18.46±10.74 | 12.28±3.81 |
| **-151** | chr5: 128965336 | 17.11±11.76 | 11.08±3.96 |
| **-179** | chr5: 128965308 | 20.53±12.42 | 15.51±5.58 |
