# Supplementary figures and images for: Downregulation of SLC27A6 by DNA Hypermethylation Promotes Proliferation but Suppresses Metastasis of Nasopharyngeal Carcinoma Through Modulating Lipid Metabolism
Source: Front Oncol. 2022 Jan 3;11:780410. doi: 10.3389/fonc.2021.780410 (PMC8761909; doi:10.3389/fonc.2021.780410)

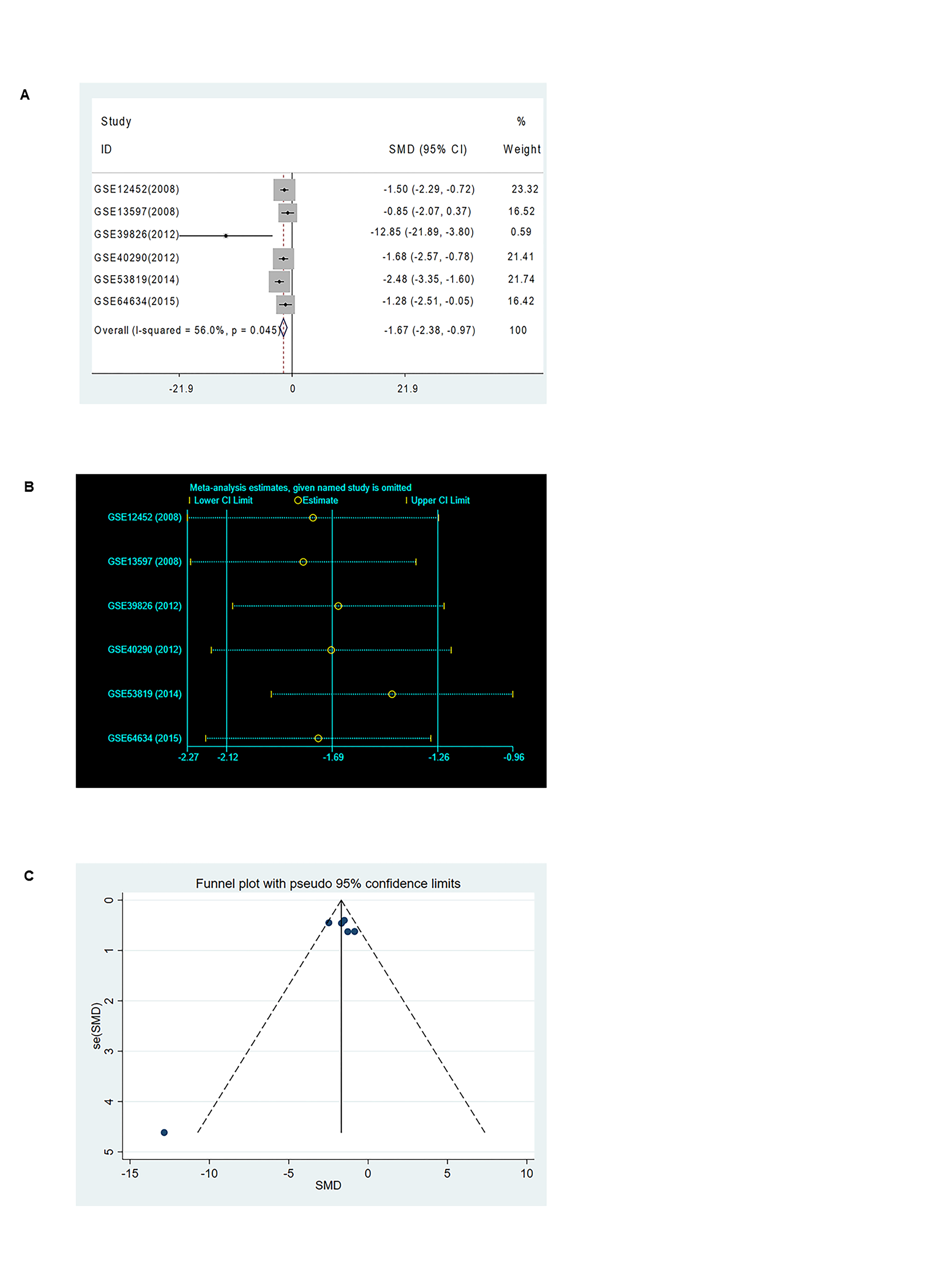

Supplement: Supplementary file 4 [file Image_1.tif]
